# Supplementary material for: Heat adaptation of phage T7 under an extended genetic code
Source: Virus Evol. 2021 Dec 1;7(2):veab100. doi: 10.1093/ve/veab100 (PMC8923235; doi:10.1093/ve/veab100)
Supplement: veab100_Supp [file veab100_supp.zip › Sequencing_Analysis.zip!/Sequencing_Analysis/S2/evidence/SNP_4.html]

BRESEQ :: Evidence


| Predicted mutation | | | | | | |
| --- | --- | --- | --- | --- | --- | --- |
| evidence | seq id | position | mutation | annotation | gene | description |
| RA | NC\_001604.1 | 18,419 | T→C | intergenic (–/–) | – / – | –/– |

| Read alignment evidence... | | | | | | | | | | | |
| --- | --- | --- | --- | --- | --- | --- | --- | --- | --- | --- | --- |
|  | seq id | position | | ref | new | freq | score (cons/poly) | reads | annotation | genes | product |
| \* | NC\_001604.1 | 18,419 | 0 | T | C | 99.2% | 17725.9 / 10.9 | 5783 | intergenic (–/–) | *–/–* | –/– |
| Reads supporting (aligned to +/- strand):  **ref** base T (16/31);  **new** base C (2912/2785);  **total** (2943/2840) | | | | | | | | | | | |
| Fisher's exact test for biased strand distribution *p*-value = 2.69e-02 | | | | | | | | | | | |
| Kolmogorov-Smirnov test that lower quality scores support variant *p*-value = 1.00e+00 | | | | | | | | | | | |

Only 100 of 5791 total aligned reads displayed.  

`GGGATTGCTGGATGGGGTGATACCGCCGAGGACTTCTTGAATAACCCGTTCATAACCGAGCCTAAAACGTCTGTGCTTAAGTCCGGTAAGAACAAAGGCCAAGAGGTTACTAAATGGGTTAAACGCGACCCTGAGCCTCATGAGACGCTTTGGGACTGCATTAAGTCCATTGGCGCGAAGGCTGGTATGACCGAAGAGGATATTATCAAGCAGGGCCAAATGGCTCGAATCCTACGGTTCAACGAGTACAACTTTATTGACAAGGAGATTTACCTGTGGAGACCGTAGCGTATATTGGTCTGGGTCTTTGTGTTCTCGGAGTGTGCCTCATTTCGTGGGGCCTTTGGGACTTAGCCAGAATAATCAAGTCGTTACACGACACTAAGTGATAAACTCAAGGTCCCTAAATTAATACGACTCACTATAGGGAGATAGGGGCCTTTACGATTATTACTTTAAGATTTAACTCTAAGAGGAATCTTTATTATGTTAACACCTATTAACCAATTACTTAAGAACCCTAACGATATTCCAGATGTACCTCGTGCAACCGCTGAGTATCTACAGGTTCGATTCAACTATGCGTACCTCGAAGCGT  >  NC_001604.1/18119‑18716`  

                                                                                                                                                                                                                                                                                                            `|`                                                                                                                                                                                                                                                                                                           
         `ggATGGGGTGATACCTCCGAGGACTTCTTGAATAACCCGTTCATAACCGAGCCTAAAACGTCTGTGCTTAAGTCCGGTAAGAACAAAGGCCAAGAGGTTACTAAATGGGTTAAACGCGACCCTGAGCCTCAGGAGACGCTTTGGGACTGCATTAAGTCCATTGGCGCGAAGGCTGGTATGACCGAAGAGGATATTATCAAGCAGGGCCAAATGGCTCGAATCCTACGGTTCAACGAGTACAACTTTATTGACAAGGAGATTTACCTGTGGAGACCGTAGCGTATATTGGTCCGGGTCtttt                                                                                                                                                                                                                                                                                                  >  1:600559/1‑300 (MQ=255)`  
         `ggATGGGGTGATACCGCCGAGGACTTCTTGAATAACCCGTTCATAACCGAGCCTAAAACGTCTGTGCTTAAGTCCGGTAAGAACAAAGGCCAAGAGGTTACTAAATGGGTTAAACGCGACCCTGAGCCTCATGAGACGCTTTGGGACTGCATTAAGTACATTGGCGCGAAGGCTGGTATGACCGAAGAGGATATTATCAAGCAGGGCCAAATGGCTCGAATCCTAAGGTTCAACGAGTACAACTTTATTGACAAGGAGATTTACCTGTGGAGACCGTAGCGTATATTGGTCCGGTTCttgt                                                                                                                                                                                                                                                                                                  >  1:723961/1‑299 (MQ=255)`  
             `ggggTGATACCGCCGAGGACTTCTTGAATAACCCGTTCATAACCGAGCCTAAAACGTCTGTGCTTAAGTCCGGTAAGAACAAAGGCCAAGAGGTTACTAAATGGGTTAAACGCGACCCTGAGCCTCATGAGACGCTTTGGGACTGCATTAAGTCCATTGGCGCGAAGGCTGGTATGACCGAAGAGGATATTATCAAGCAGGGCCAAATGGCTCGAATCCTACGGTTCAACGAGTACAACTTTATTGACAAGGAGATTTACCTGTGGATACCGTAGCGTCTATTGGTCCGGGTCTTTGTGtt                                                                                                                                                                                                                                                                                              >  1:107812/1‑301 (MQ=255)`  
              `gggTGATACCGCCGAGGACTTCTTGAATAACCCGTTCATAACCGAGCCTAAAACGTCTGTGCTTAAGTCCGGTAAGAACAAAGGCCAAGAGGTTACTAAATGGGTTAAACGCGACCCTGAGCCTCATGAGACGCTTTGGGACTGCATTAAGTCCATTGGCGCGAAGGCTGGTATGACCGAAGAGGATATTATCAAGCAGGGCCAAATGGCTCGAATCCTACGGTTCAACGAGTACAACTTTATTGACAAGGAGATTTACCTGTGGAGACCGTAGCTTATATTGGTCCGGGTCTTTGTGttt                                                                                                                                                                                                                                                                                             >  1:187246/1‑300 (MQ=255)`  
              `gggTGATACCGCCGAGGACTTCTTGAATAACCCGTTCATAACCGAGCCTAAAACGTCTGTGCTTAAGTCCGGTAAGAACAAAGGCCAAGAGGTTACTAAATGGGTTAAACGCGACCCTGAGCCTCATGAGACGCTTTGGGACTGCATTAAGTCCATTGGCGCGAAGGCTGGTATGACCGAAGAGGATATTATCAAGCAGGGCCAAATGGCTCGAATCCTACGGTTCAACGAGTACAACTTTATTGACAAGGAGATTTACCTGTGGAGACCGTAGCGTATATTGGTCCGGGTCttttttttt                                                                                                                                                                                                                                                                                             >  1:804295/1‑295 (MQ=255)`  
                                `cttcttGAATAACCCGTTCATAACCGAGCCTAAAACGTCTGTGCTTAAGTCCGGTAAGAACAAAGGCCAAGAGGTTACTAAATGGGTTAAACGCGACCCTGAGCCTCATGAGACGCTTTGGGACTGCATTAAGTCCATTGGCGCGAAGGCTGGTATGACCGAAGAGGATATTATCAAGCAGGGCCAAATGGCTCGAATCCTACGGTTCAACTAGTACACCTTTATTGACAAGGAGATTTCCCTGTGGGGACCGTAGCGTATATTGGTCCGGGTCTTTGTGTTATCGTAGTGTGCCtacttt                                                                                                                                                                                                                                                                           >  1:56800/1‑296 (MQ=255)`  
                                 `ttcttGAATAACCCGTTCATAACCGAGCCTAAAACGTCTGTGCTTAAGTCCGGTAAGAACAAAGGCCAAGAGGTTACTAAATGGGTTAAACGCGACCCTGAGCCTCATGAGACGCTTTGGGACTGCATTAAGTCCATTGGCGCGAAGGCTGGTATGACCGAAGAGGATATTATCAAGCAGGGCCAAATGGCTCGAATCCTACGGTTCAACGAGTACAACTTTATTGACAAGGAGATTTACCTGTGGAGAACGTAGCGTATATTGGTCCGGGTCTTTGTGTTCTCGGAGTGTGCCTCAtttt                                                                                                                                                                                                                                                                          >  1:672882/1‑300 (MQ=255)`  
                                      `gAATAACCCGTTCATAACCGAGCCTAAAACGTCTGTGCTTAAGTCCGTTAAGAACAAAGGCAAAGAGGTTACTTAATGGGTTAAACGCGACCCTGACCCCCATGAGACGCTTGGGGACGGCATTAAGTCCATTGGCGCGAAGGCTGGTATGACCGAAGAGGATATTATCAACCAGGGCAAAATGGCTCGAATCCTACGGTTCAACGAGTACACCTTTATTGACAAGGAGATTACCCTGTGAAGCCCGTAGCGTAAATGGGTCGGGGCTTTTGTGTTCTCGGAGGGGGCCCCTTTTctgccc                                                                                                                                                                                                                                                                     >  1:64965/1‑296 (MQ=255)`  
                                      `gAATAACCCGTTCATAACCGAGCCTAAAACGTCTGTGCTTAAGTCCGGTAAGAACAAAGGCCAAGAGGTTACTAAATGGGTTAAACGCGACCCTGAGCCTCATGAGACGCTTTGGGACTGCATTAAGTCCATTGGCGCGAAGGCTGGTATGACCGAAGAGGATATTATCAAGCAGGGCCAAATGGCTCGAATCCTACGGTTCAACGAGTACAACTTTATTGACAAGGAGATTTACCTGTGGAGACCGTAGCGTATATTGGTCCGGGTCTTTGTGTTCTCGGAgggctgtctcttatacaca                                                                                                                                                                                                                                                                     >  1:391638/1‑283 (MQ=255)`  
                                               `gTTCATAACCGAGCCTAAAACGTCTGTGCTTAAGTCCGGTAAGAACAAAGGCCAAGAGGTTACTAAATGGGTTAAACGCGACCCTGAGCCTCATGAGACGCTTTGGGACTGCATTAAGTCCATTGGCGCGAAGGCTGGTATGACCGAAGAGGATATTATCAAGCAGGGCCAAATGGCTCGAATCCTACGGTTCAACGAGTACAACTTTATTGACAAGGAGATTTACCTGTGGAGACCGTAGCGTATATTGGTCCGGGTCTTTGTGTTCTCGGAGTGTGCCTCATTTCGTGGGGCCTTTggg                                                                                                                                                                                                                                                            >  1:609378/1‑301 (MQ=255)`  
                                                           `gCCTAAAACGTCTGTGCTTAAGTCCGGTAAGAACAAAGGCCAAGAGGTTACTAAATGGGTTAAACGCGACCCTGAGCCTCATGAGACGCTTTGGGACTGCATTAAGTCCATTGGCGCGAAGGCTGGTATGACCGAAGAGGATATTATCAAGCAGGGCCAAATGGCTCGAATCCTACGGTTCAACGAGTACAACTTTATTGACAAGGAGATTTACCTGTGGAGACCGTAGCGTATATTGGTCCGGGTCTTTGTGTTCTAGGAGTGTGCCTCATTTCGTGGGGCCTTTGGGACTTAGCCAGaa                                                                                                                                                                                                                                                >  1:172278/1‑301 (MQ=255)`  
                                                            `ccTAAAACGTCTGTGCTTAAGTCCGGTAAGAACAAAGGCCAAGAGGTTACTAAATGGGTTAAACGCGACCCTGAGCCTCATGAGACGCTTTGGGACTGCATTAAGTCCATTGGCGCGAAGGCTGGTATGACCGAAGAGGATATTATCAAGCATGGCCAAATGGCTCGAATCCTACGGTTCAACGAGTACAACTTTATTGACAAGGAGATTTACCTGTGGAGACCGTAGCGTATATTGGTCCGGGTCTTTGTGTTCTCGGAGTGTGCCTCATTTCGTGGGGCCTTTGGGACTTAGcccaaat                                                                                                                                                                                                                                               >  1:121797/1‑296 (MQ=255)`  
                                                                `aaaCGTCTGTGCTTAAGTCCGATAAGAACAAAGGCCAAGAGGTTACTAAATGGGTTAAACGCGACCCTGAGCCTCATGAGACGCTTTGGGACTGCATTAAGTCCATTGGCGCGAAGGCTGGTATGACCGAAGAGGATATTATCAAGCAGGGCCAAATGGCTCGAATCCTACGGTTCAACGAGTACAACTTTATTGACAAGGAGATTTACCTGTGGAGACCGTAGCGTATATTGGTCCGGGTCTTTGTGTTCTCGGAGTGTGCCTCATTTCGTGGGGCCTTTGGGACTTAGCCAGAATAATc                                                                                                                                                                                                                                           <  1:416444/301‑1 (MQ=255)`  
                                                                        `gtgCTTAAGTCCGGTAAGAACAAAGGCCAAGAGGTTACTAAATGGGTTAAACGCGACCCTGAGCCTCATGAGACGCTTTGGGACTGCATTAAGTCCATTGGCGCGAAGGCTGGTATGACCGAAGAGGATATTATCAAGCAGGGCCAAATTGCTCGAATCCTACGGTTCAACGAGTACAACTTTATTGACAAGGAGATTTACCTGTGGAGACCGTAGCGTATATTGGTCCGGGTCTTTGTGTTCTCGGAGTGTGCCTCATTTCGTGGGGCCTTTGGGACTTAGCCAGAATAATCAAGTCGtt                                                                                                                                                                                                                                   >  1:315665/1‑301 (MQ=255)`  
                                                                        `gtgCTTAAGTCCGGTAAGAACAAAGGCCAAGAGGTTACTAAATGGGTTAAACGCGACCCTGAGCCTCATGAGACGCTTTGGGACTGCATTAAGTCCATTGGCGCGAAGGCTGGTATGACCGAAGAGGATATTATCAAGCAGGGCCAAATGGCTCGAATCCTACGGTTCAACGAGTACAACTTTATTGACAAGGAGATTTACCTGTGGAGACCGTAGCGTATATTGGTCCGGGTCTTTGTGTTCTCGGAGTGTGCCTCATTTCGTGGGGCCTTTGGGACTTAGCCAGAATAATCAAGTCGtt                                                                                                                                                                                                                                   >  1:753234/1‑301 (MQ=255)`  
                                                                        `gtgCTTAAGTCCGGTAAGAACAAAGGCCAAGAGGTTACTAAATGGGTTAAACGCGACCCTGAGCCTCATGAGACGCTTTGGGACTGCATTAAGTCCATTGGCGCGAAGGCTGGTATGACCGAAGAGGATATTATCAAGCAGGGCCAAATGGCTCGAATCCTACGGTTCAACGAGTACAACTTTATTGACAAGGAGATTTACCTGTGGAGACCGTAGCGTATATTGGTCCGGGTCTTTGTGTTCTCGGAGTGTGCCTCATTTCGTGGGGCCTGTGGGAcgctgtctcttatacaaatctccg                                                                                                                                                                                                                                   >  1:64197/1‑278 (MQ=255)`  
                                                                               `aGTCCGGTAAGAACAAAGGCCAAGAGGTTACTAAATGGGTTAAACGCGACCCTGAGCCTCATGAGACGCTTTGGGACTGCATTAAGTCCATTGGCGCGAAGGCTGGTATGACCGAAGAGGATATTATCAAGCAGTGCCAAATGGCTCGAATCCTTCGGTTCAACGAGTACAACTTTATTGACAAGGAGATTTACCTGTGGAGACCGTAGCGTATATTGGTCCGGGTCTTTGTGTTCTCGGAGTGTGCGTCATTTCGTGGGGCCTTTGGGACTTAGCCCGAATAATCCTGTCGTTACACGac                                                                                                                                                                                                                            >  1:777427/1‑301 (MQ=255)`  
                                                                                 `tCCGGTAAGAACAAAGGCCAAGAGGTTACTAAATGGGTTAAACGCGACCCTGAGCCTCATGAGACGCTTTGGGACTGCATTAAGTCCATTGGCGCGAAGGCTGGTATGACCGAAGAGGATATTATCAAGCAGGGCCAAATGGCTCGAATCCTACGGTTCAACGAGTACAACTTTATTGACAAGGAGATTTACCTGTGGAGACCGTAGCGTATATTGGTCCGGGTCTTTGTGTTCTCGGAGTGTGCCTCATTTCGTGGGGCCTTTGGGACTTAGCCAGAATAATCAAGTCGTTACACGacac                                                                                                                                                                                                                          <  1:194098/301‑1 (MQ=255)`  
                                                                                    `ggTAAGAACAAAGGCCAAGAGGTTACTAAATGGGTTAAACGCGACCCTGAGCCTCATGAGACGCTTTGGGACTGCATTAAGTCCATTGGCGCGAAGGCTGGTATGACCGAAGAGGATATTATCAAGCAGGGCCAAATGGCTCGAATCCTACGGTTCAACGAGTACAACTTTATTGACAAGGAGATTTACCTGTGGAGACCGTAGCGTATATTGGTCCGGGTCTTTGTGTTCTCGGAGTGTGCCTCATTTCGTGGGGCCTTTGGGACTTAGCCAGAATAATCAAGTCGTTACACGACACTaa                                                                                                                                                                                                                       >  1:538260/1‑301 (MQ=255)`  
                                                                                      `caggAACAAAGGCCAAGAGGTTACTAAATGGGTTAAACGCGATCCTGAGCCTCATGAGTCGCTTTGGGACTGCATTAAGTCCATTGGCGCGAAGGCTGGTATGACCGAAGAGGATAGTATCAAGCAGGGCCAAATGGCTCGAATCCTACGGTTCAACGAGTACAACTTTATTGCCAAGGAGATTTACCTGTGGAGACCGTAGCGTATATTGGTCCGGGTCTTTGTGTTCTCGGAGTGTGCCTCATTTCGTGGGGCCTTTGGGACTTAGCCAGAATAATCAAGTCGTTACACGACACTAAGt                                                                                                                                                                                                                     <  1:100489/298‑1 (MQ=255)`  
                                                                                        `aGAACAAAGGCCAAGAGGTTACTAAATGGGTTAAACGCGACCATGAGCCTCATGAGACGCTTGGGGACTGCATTAAGTCCATTGGCGCGAAGGCTGGAATGACCGAAGAGGATATTATCAAGCAGGTCCAAATGGCTCGAATCCTACGGTTCAACGAGTACAACTTTAATGACAAGGAGATTTACCTGTGGAGACCGTAGCGTATATTGGTCCGGGTCTTTGTGTTCTCGGAGTGTGCCTCATTTCGTGGGGCCTTTGGGACTTAGCCAGAATAATCAAGTCGTTACACGACACTAAGTGa                                                                                                                                                                                                                   <  1:323475/301‑1 (MQ=255)`  
                                                                                         `gAACAAAGGCCAAGAGGTTACTAAATGGGTTAAACGCGACCCTGAGCCTCATGAGACGCTTTGGGACTGCATTAAGTCCATTGGCGCGAAGGCTGGTATGACCGAAGAGGATATTATCAAGCAGGGCCAAATGGCTCGAATCCTACGGTTCAACGAGTACAACTTTATTGACAAGGAGATTTACCTGTGGAGACCGTAGCGTATATTGGTCCGGGTCTTTGTGTTCTCGGAGTGTGCCTCATTTCGTGGGGCCTTTGGGACTTAGCCAGAATCATCAAGTCGTTACACGACACTAAGtctg                                                                                                                                                                                                                  >  1:594412/1‑298 (MQ=255)`  
                                                                                             `aaaGGCCAAGAGGTTACTAAATGGGTTAAACGCGACCCTGAGCCTCATGAGACGCTTTGGGACTGCATTAAGTCCATTGGCGCGAAGGCTGGTATGACCGAAGAGGATATTATCAAGCAGGGCCAAATGGCTCGAATCCTACGGTTTAACGAGTACAACTTTAGTGACAAGGAGATTTACCTGTGGAGACCGTAGCGTATATTGGTCCGGGTCTTTGTGTTCTCGGAGTGTGCCTCATTTCGTGGGGCCTTTGGGACTTAGCCAGAATAATCAAGTCGTTACACGACACTAAGTGATAAAc                                                                                                                                                                                                              <  1:826716/301‑1 (MQ=255)`  
                                                                                              `aaGGCCAAGAGGTTACTAAATGGGTTAAACGCGACCCTGAGCCTCATGAGACGCTTTGGGACTGCATTAAGTCCATTGGCGCGAAGGCTGGTATGACCGAAGAGGATATTATCAAGCAGGGCCAAATGGCTCGAATCCTACGGTTCAACGAGTACAACTTTATTGACAAGGAGATTTACCTGTGGAGACCGTAGCGTATATTGGTCCGGGTCTTTGTGTTCTCGGAGTGTGCCTCATTTCGTGGGGCCTTTGGGACTTAGCCAGAATAATCAAGTCGTTACACGACACTAAgctgtctctt                                                                                                                                                                                                             >  1:145384/1‑292 (MQ=255)`  
                                                                                               `aGGCCAAGAGGTTACTAAATGGGTTAAACGCGCCCCTGAGCCTCATGAGACGCTTTGGGACTGCATTAAGTCCATTGGCGCGAAGGCTGGTATGACCGAAGAGGATATTATCAAGCAGGGCCAAATGGCTCGAATCCTACGGTTCAACGAGTACAACTTTATTGACAAGGAGATTTACCTGTGGAGACCGTAGCGTATATTGGTCCGGGTCTTTGTGTTCTCGGAGTGTGCCTCATTTCGTGGGGCCTTTGGGACTTAGCCAGAATAATCAAGTCGTTACACGACACTAAGTGATAAACTc                                                                                                                                                                                                            <  1:682924/301‑1 (MQ=255)`  
                                                                                               `aGGCCAAGAGGTTACTAAATGGGTTAAACGCGACCCTGAGCCTCATGAGACGCTTTGGGACTGCATTAAGTCCATTGGCGCGAAGGCTGGTATGACCGAAGAGGATATTATCAAGCAGGGCCAAATGGCTCGAATCCTACGGTTCAACGAGTACAACTTTATTGACAAGGAGATTTACCTGTGGAGACCGTAGCGTATATTGGTCCGGGTCTTTGTGTTCTCGGAGTGTGCCTCATTTCGTGGGGCCTTTGGGACTTAGCCAGAATAATCAAGTCGTTACACGACACTAAGTGATAAACTc                                                                                                                                                                                                            <  1:113886/301‑1 (MQ=255)`  
                                                                                                    `aaGAGGTTACTAACTGGTTTAAACTCGACCCTGAGCCTCATGAGACGCGTTGGGACTGCGTTAAGTCCATTGGCGCGAAGGCTGGTTTGACCGAAGAGGATATTATCAAGCAGGGCCAAATGGCTCGAATCCTACGGTTCAACGAGTACAACTTTATTGACAAGGGTATTTACCTGTGGAGACCGTAGCGTATATTGGTCCGGGTCTTTGTGTTCTCGGAGTGTGCCTCATTTCGTGGGGCCTTTGGGACTTAGCCAGAATAATCAAGTCGTTACACGACACTAAGTGATAAACTCAAGGt                                                                                                                                                                                                       <  1:441465/301‑1 (MQ=255)`  
                                                                                                      `gagGTTACTAAATGGGTTAAACGCGACCCTGAGCCTCATGAGACGCTTTGGGACTGCATTAAGTCCATTGGCGCGAAGGCTGGTATGACCGAAGAGGATATTATCAAGCAGGGCCAAATGGCTCGAATCCTACGGTTCAACGAGTACAACTTTATTGACAAGGAGATTTACCTGTGGAGACCGTAGCGTATATTGGTCCGGGTCTTTGTGTTCTCGGAGTGTGCCTAATTTCGTGGGGCCTTTGGGACTTAGCCAGAATAATCAAGTCGGTACACGACACTAAGtcgtgtaacgacttgat                                                                                                                                                                                                     >  1:343645/1‑285 (MQ=255)`  
                                                                                                       `agGTTACTAAATGGGTTAAACGCGACCCTGAGCCTCATGAGACGCTTTGGGACTGCATTAAGTCCATTGGCGCGAAGGCTGGTATGACCGAAGAGGATATTATCAAGCAGGGCCAAATGGCTCGAATCCTACGGTTCAACTAGTACAACTTTATTGACAAGGAGATTTACCTGTGGAGACCGTAGCGTATATTGGTCCGGGTCTTTGTGTTCTCGGAGTGTGCCTCATTTCGTGGGGCCTTTGGGACTTAGCCAGAATAATCAAGTCGTTACACGACACTAAGTGATAAACTCAAGGTccc                                                                                                                                                                                                    >  1:530485/1‑301 (MQ=255)`  
                                                                                                             `cTAAATGGGTTAAACGCGACCCTGAGCCTCATTAGACGCTTTGGGACTGCATTAAGTCCATTGGCGCGAAGGCTGGTATGACCGAAGAGGATATTATCAAGCAGGGCCAAATGGCTCGAATCCTACGGTTCAACGAGTACAACTTTATTGACAAGGAGATTTACCTGTGGAGACCGTAGCGTATATTGGTCCGGGTCTTTGTGTTCTCGGAGTGTGCCTCATTTCGTGGGGCCTTTGGGACTTAGCCAGAATAATCAAGTCGTTACACGACACTAAGTGATAAACTCAAGGTCCCTAAAtt                                                                                                                                                                                              >  1:690130/1‑301 (MQ=255)`  
                                                                                                                 `aTGGGTTAAACGCGACCCTGAGCCTCAGGAGACGCGTTGGGACTGCATTAAGTCCATTGGCGCGAAGGCTGGTATGACCGAAGAGGATATTATCAAGCAGGGCCAAATGGCTCGAATCCTACGGTTCAACGAGTACAACTTTATTGACAAGGAGATTTACCTGTGGAGACCGTAGCGTATATTGGTCCGGGTCTTTGTGTTCTCGGAGTGTGCCTCATTTCGTGGGGCCTTTGGGACTTAGCCAGAATAATCAAGTCGTTACACGACACTAAGTGATAAACTCAAGGTCCCTAAATTAATa                                                                                                                                                                                          <  1:634386/301‑1 (MQ=255)`  
                                                                                                                  `agggTTAAACGCGACCCTGAGGCTCATGAGACGCTTTGGGACTGCATTAAGTCCATTGGCGCGAAGGCTGGTATGACCGAAGAGGATATTATCAAGCAGGGTCAAATGGCTCGAATCCTACGGTTCAACGAGTACAACTTTATTGACAAGGAGATTTACCTGTGGAGACCGTAGCGTATATTGGTCCGGGTCTTTGTGTTCTCGGAGTGTGCTTCATTTCGTGGGGCCTTTGGGACTTAGCCAGAATAATCAAGTCGTTACACGACACTAAGTGATAAACTCAGGGTCCCTAAATTAATAc                                                                                                                                                                                         <  1:301737/300‑1 (MQ=255)`  
                                                                                                                    `ggTTAAACGCGACCCTGAGCCTCATGAGACGCTTTGGGACTGCATTAAGTCCATTGGCGCGAAGGCTGGTATGACCGAAGAGGATATTATCAAGCAGGGCCAAATGGCTCGAATCCTACGGTTCAACGAGTACAACTTTATTGACAAGGAGATTTACCTGTGGAGACCGTAGCGTATATTGGTCCGGGTCTTTGTGTTCTCGGAGTGTGCCTCATTTCGTGGGGCCTTTGGGACTTAGCCAGAATAATCAAGTCGTTACACGACACTAAGTGATAAACTCAAggctgtctcttatacacat                                                                                                                                                                                       >  1:272283/1‑284 (MQ=255)`  
                                                                                                                        `aaaCGCGACCCTGAGCCTCATGAGACGCTTTGGGACTGCATTAAGTCCATTGGCGCGAAGGCTGGTATGACCGAAGAGGATATTATCAAGCAGGGCCAAATGGCTCGAATCCTACGGTTCAACGAGTACAACTTTATTGACAAGGAGATTTACCTGTGGAGACCGTAGCGTATATTGGTCCGGGTCTTTGTGTTCTCGGAGTGTGCCTCATTTCGTGGGGCCTTTGGGACTTAGCCAGAATAATCAAGTCGTTACACGACACTAAGTGATAAACTCAAGGTCCCTAAATTAATACGACTCa                                                                                                                                                                                   >  1:56019/1‑301 (MQ=255)`  
                                                                                                                          `aCGCGACCCTGAGCCTCATGAGACGCTTTGGGACTGCATTAAGTCCATTGGCGCGAAGTCTGGTATGACCGAAGAGGATATTATCAAGCAGGGCCAAATGGCTCGAATCCTACGGTTCAACGAGTACAACTTTATTGACAAGGAGATTTACCTGTGGAGACCGTAGCGTATATTGGTCCGGGTCTTTGTGTTCTCGGAGTGTGCCTCATTTCGTGGGGCCTTTGGGACTTAGCCAGAATAATCAAGTCGTTACACGACACTAAGTGATAAACTCAAGGTCCCTAAATTAATACGACTCACt                                                                                                                                                                                 >  1:371175/1‑301 (MQ=255)`  
                                                                                                                               `aCCCTGAGCCTCATGAGACGCTTTGGGACTGCATTAAGTCCATTGGCGTGAAGGCTGGTATGACCGAAGAGGATATTATCAAGCAGGGCCAAATGGCTCGAATCCTACGGTTCAACGAGTACAACTTTATTGACAAGAAGATTTACCTGTGGAGACCGTAGCGTATATTGGTCCGGGTCTTTGTGTTCTCGGAGTGTGCCTCATTTCGTGGGGCCTTTGGGACTTAGCCAGAATAATCAAGTCGTTACACGACACTAAGTGATAAACTCAAGGTCCCTAAATTAATACGACTCACTATAgg                                                                                                                                                                            >  1:329657/1‑301 (MQ=255)`  
                                                                                                                               `aCCCTGAGCCTCATGAGACGCTTTGGGACCGCATTAAGTCCATTGGCGCGAAGGCTGGTATGACCGAAGAGGATATTATCAAGCAGGGCCAAATGGCTCGAATCCTACGGTTCAACGAGTACAACTTTATTGACAAGGAGATTTACCTGTGGAGACCGTAGCGTATATTGGTCCGGGTCTTTGTGTTCTCGGAGTGTGCCTCATTTCGTGGGGCCTTTGGGACTTAGCCAGAATAATCAAGGCGTTACACGACACTAAGTGATAAACTAAAGGGCACTAAATTAATACGACTCACGATAgg                                                                                                                                                                            >  1:545426/1‑301 (MQ=255)`  
                                                                                                                                `cccTGAGCCTCATGAGACGCTTTGGGACTGCATTAGGTCCATTGGCGCGAAGGCTGGTATGACCGAAGAGGATATTATCAAGCAGGGCCAAATGGCTCGAATCCTACGGTTCAACGAGTACAACTTTATTGACAAGGAGATTTACCTGTGGAGACCGTAGCGTATATTGGTCCGGGTCTTTGTGTTCTCGGAGTGTGCCTCATTTCGTGGGGCCTTTGGGACTTAGCCAGAATAATCAAGTCGTTACACGACACTAAGTGATAAACTCAAGGTCCCTAAATTAATACGACTCACTATAggg                                                                                                                                                                           <  1:279972/301‑1 (MQ=255)`  
                                                                                                                                `cccTGAGCCTCATGAGACGCTTTGGGACTGCATTAAGTCCATTGGCGCGAAGGCTGGTATGACCGAAGAGGATATTATCAAGCAGGGCCAAATGGCTCGAATCCTACGGTTCAACGAGTACAACTTTATTGACAAGGAGATTTACCTGTGGAGACCGTAGCGTATATTGGTCCGGGTCTTTGTGTTCTCGGAGTGTGCCTCATTTCGTGGGGCCTTTGGGACTTAGCCAGACTAATCAAGTCGTTACACGACACTAAGTGATAAACTCAAGGTCCCTAAATTAGTACGACTCACTATAgag                                                                                                                                                                           >  1:581310/1‑299 (MQ=255)`  
                                                                                                                                `cccTGAGCCTCATGAGACGCTTTGGGACTGCATGAAGTCCATTGGCGCGAAGGCTGGTATGACCGAAGAGGATATTATCAAGCAGGGCCAAATGGCTCGAATCCTACGGTTCAACGAGTACAACTTTATTGACAAGGAGATTTACCTGTGGAGACCGTAGCGTATATTGGTCCGGGTCTTTGTGTTCTCGGAGTGTGCCTCATTTCGTGGGGCCTTTGGGACTTAGCCAGAATAATCAAGTCGTTACACGACACTAAGTGATAAACTCAAGGTCCCTAAATTAATACGACTCACTATAggg                                                                                                                                                                           <  1:86854/301‑1 (MQ=255)`  
                                                                                                                                `cccTGAGCCTCATGAGACGCTTGGGGACTGAATTAAGTCAATGGGCGCGAAGGCTGGTATGACCGAATAGTCCATTATCAAGCAGGGCAAACTGGCTCGAATCCGACTTTTCAACGAGTAAAACTTTATTGACAAGGAGATTTACCTGTGGAGACCGTAGCGTATATTGGTCCGGGTTTTTGTGTTCTCGGAGTGTGCCTCATTTCGTGGGGCCTTTGGGACTTAGCCAGAATAATCAAGTCGTTACACGACACTAAGTGATAAACTCAAGGTCCCTAAATTAATACGACTCACTATAggg                                                                                                                                                                           <  1:166645/301‑1 (MQ=255)`  
                                                                                                                                 `ccTGAGCCTCATGAGACGCTTTGGGACTGCATTAAGTCCATTGGCGCGAAGGCTGGTATGACCGAAGAGGATATTATCAAGCAGGGCCAACTGGGTCGAATCCTACGGTTCAACGAGTACAACTTTATTGACAAGGAGATTTATCTGTGGAGACCGTAGCGTATATTGGTCCGGGTCTTTGTGTTCTCGGAGTGTGCCTCATTTCGTGGGGCCTTTGGGACTTAGCCAGAATAATCAAGTCGTTACACGACACTAAGTGATAACCTCAAGGTCCCTAAATTAATACGACTCACTATAGGga                                                                                                                                                                          <  1:697340/301‑1 (MQ=255)`  
                                                                                                                                 `ccTGAGCCTCATGAGACGCTTTGGGACTGCATTAAGTCCATTGGCGCGAAGGCTGGTATGACCGAAGAGGATATTATCAAGCAGGGCCAAATGGCTCGAATCCTACGGTTCAACGAGTACAACTTTATTGACAAGGAGATTTACCTGTGGAGACCGTAGCGTATATTGGTCCGGGTCTTTGTGTTCTCGGAGTGTGCCTCATTTCGTGGGGCCTTTGGGACTTAGCCAGAATAATCAAGTCGTTACACGACACTAAGTGATAAACTCAAGGTCCCTAAATTAATACGACTCACTATAGGga                                                                                                                                                                          <  1:587789/301‑1 (MQ=255)`  
                                                                                                                                 `ccTGAGCCGCATGAATCGCTTTGGGAATGCATTAAGTCCAGTGGCGAGGAGGCTGGTATGCCCGCAGAGGACATTATCAAGCAGAGCAAAATGGCTCGAATCCTACGGTTCAACGAGTACAACTTGATGGACAAGGAGACTTACTATTGGAGACCGGAGCGGAGATTGGTCCGTGTCTATGTATTCTCGGAGTGTGCCTAATTTCGTGGGGCATTTGGGAGGTAGCCAGAATAATCAAGTAGTGACACGACACGAAGTGATAAAATCAAGGTAACTAAATTAATACGACTCACTATAGGga                                                                                                                                                                          <  1:309130/301‑1 (MQ=255)`  
                                                                                                                                 `acagAGCCTCATGAGACGCTTTGGGACTGCATTAAGTCCATTGGCGCGAAGGCTGGTATAACCGAAGAGGATATTATCAAGCAGGGCCAAATGGCTCGAATCCTACGGTTCAACGAGTACAACTTTATTGACAAGGAGATTTACCTGTGGAGACCGTAGCGTATATTGGTCCGGGTCTTTGTGTTCTCGGAGTGTGCCTCATTTCGTGGGGCCTTTGGGACTTAGCCAGAATAATCAAGTCGTTACACGACACTAAGTGATAAACTCAAGGTCCCTAAATTAATACGACTCACTATAGGga                                                                                                                                                                          <  1:51342/298‑1 (MQ=255)`  
                                                                                                                                 `aagagacagcATGAGACGCTTTGGGACTGCATTAAGTCCATTGGCGCGAAGGCTGGTATGACCGAAGAGGATATTATCAAGCAGGGCCAAATGGCTCGAATCCTACGGTTCAACGAGTACAACTTTATTGACAAGGAGATTTACCTGTGGAGACCGTAGCGTATATTGGTCCGGGTCTTTGTGTTCTCGGAGTGTGCCTCATTTCGTGGGTCCTTTGGGACTTAGCCAGAATAATCAAGTCGTTACACGACACTAAGTGATAAACTCAAGGTCCCTAAATTAATACGACTCACAATAGGga                                                                                                                                                                          <  1:179340/292‑1 (MQ=255)`  
                                                                                                                                  `cTGAGCCTCATGAGACGCTTTGGGACTGTATTAAGTCCATTGGCGCGAAGGCTGGTATGACCGAAGAGGATATTATCAAGCAGGGCCAAATGGCTCGAATCCTACGGTTCAACGAGTACAACTTTATTGACAAGGAGATTTACCTGTGGAGACCGTAGCGTATATTGGTCCGGGTCTTTGTGTTCTCGGAGTGTGCCTCATTTCGTGGGGCCTTTGGGACTTAGCCAGAATAATCAAGTCGTTACACGACACTAAGTGATAGACTCAAGGTCCCTAAATTAATACGACTCACTATAGGgag                                                                                                                                                                         <  1:625968/301‑1 (MQ=255)`  
                                                                                                                                    `gAGCCTCATGAGACGCTTTGGGACTGCATTAAGTCCATTGGCGCGAAGGCTGGTATGACCGAAGAGGATATTATCAAGCAGGGCCAAATGGCTCGAATCCTACGGTTCAACGAGTACAACTTTATTGACAAGGAGATTTACCTGTGGAGACCGTAGCGTATATTGGTCCGGGTCTTTGTGTTCTCGGAGTGTGCCTCATTTCGTGGGGCCTTTGGGACTTAGCCAGAATAATCAAGTCGTTACACGACACTAAGTGATAAACTCAAGGTCCCTAAATTAATACGACTCACTATAGGGAGAt                                                                                                                                                                       >  1:409094/1‑301 (MQ=255)`  
                                                                                                                                         `tCATGAGACGCTTTGGGACTGCATTAAGTCCATTGGCGCGAAGGCTGGTATGACCGAAGAGGATATTATCAAGCAGGGCCAAATGGCTCGAATCCTACGGTTCAACGAGTACAACTTTATTGACAAGGAGATTTACCTGTGGAGACCGTAGCGTATATTGGTCCGGGTCTTTGTGTTCTCGGAGTGTGCCTCATTTCGTGGGGCCTTTGGGACTTAGCCAGAATAATCAAGTCGTTACACGACACTAAGTGATAAACTCAAGGTCCCTAAATTAATACGACTCACTATAGGGAGATAgggg                                                                                                                                                                  <  1:760437/301‑1 (MQ=255)`  
                                                                                                                                                   `cTTTGGGACTGCATTAAGTCCATTGGCGCGAAGGCTGGTATGACCGAAGAGGATATTATCAAGCAGGGCCAAATGGCTCGAATCCTACGGTTCAACGAGTACAACTTTATTGACAAGGAGATTTACCTGTGGAGACCGTAGCGTATATTGGTCCGGGTCTTTGTGTTCTCGGAGTGTGCCTCATTTCGTGGGGCCTTTGGGACTTAGCCAGAATAATCAAGTCGTTACACGACACTAAGTGATAAACTCAAGGTCCCTAAATTAATACGACTCACTATAgggctgtctcttatacccatct                                                                                                                                                        >  1:492859/1‑282 (MQ=255)`  
                                                                                                                                                   `cTTTGGGACTGCATTAAGTCCATTGGCGCGAAGGCTGGTATGACCGAAGAGGATATTATCAAGCAGGGCCAAATGGCTCGAATCCTACGGTTCAACGAGTACAACTTTATTGACAAGGAGATTTACCTGTGGAGACCGTAGCGTATATTGGTCCGGGTCTTTGTGTTCTCGGAGTGTGACTCATTTCGTGGGGCCTTTGGGACTTAGCCAAAAAAATCAAGTCGTTAAACGAACTTAAGTGTTAAACTAAAGGTCCCTAAATTATAAAGACCAACTAAAGGGGGTTAGGGGCCTTTAGGat                                                                                                                                                        >  1:714486/1‑301 (MQ=255)`  
                                                                                                                                                   `cTTTGGGACTGCATTAAGTCCATTGGCGCGAAGGCTGGTATGACCGAAGAGGATATTATCAAGCAGGGCCAAATGGCTCGAATCCTACGGTTCAACGAGTACAACTTTATTGACAAGGAGATTTACCTGTGGAGACCGTAGCGTATATTGGTCCGGGGCTTTGTGTTCTCGGAGTGTGCCTCATTTCGTGGGGCCTTTGGGACTTAGCCAGAATAATCAAGTCGTTACACGACACTAAGTGCTAAACTCAAGGTCCCTAAATTAATACGACTCACTTTAGGGAGATAGGGGCCTTTACGat                                                                                                                                                        >  1:129085/1‑301 (MQ=255)`  
                                                                                                                                                     `ttGGGACTGCATTAAGTCCATTGGCGCGAAGGCTGGTATGACCGAAGAGGATATTATCAAGCAGGGCCAAATGGCTCGAATCCTACGGTTCAACGAGTACAACTTTATTGACAAGGAGATTTACCTGTGGAGACCGTAGCGTATATTGGTCCGGGTCTTTGTGTTCTCGGAGTGTGCCTCATTTCGTGGGGCCTTTGGGACTTAGCCAGAATAATCAAGTCGTTACACGACACTAAGTGATAAACTCAAGGTCCCTAAATTAATACGACTCACTATAGGGAGATAGGGGCCTTTACGatta                                                                                                                                                      <  1:158419/301‑1 (MQ=255)`  
                                                                                                                                                       `gggACTGCATTAAGTCCATTGGCGCGAAGGCTGGTATGACCGAAGAGGATATTATCAAGCAGGGCCAAATGGCTCGAATCCTACGGTTCAACGAGTACAACTTTATTGACAAGGAGATTTACCTGTGGAGACCGTAGCGTATATTGGTCCGGGTCTTTGTGTTCTCGGAGTGTGCCTCATTTCGTGGGGCCTTTGGGACTTAGCCAGAATAATTAATTCGTTACACGACACTAAGTGATAAACTCAAGGTCCCTAAATTAATACGACTCACTATAGGGAGATAGGGGccctgtctcttata                                                                                                                                                    >  1:256629/1‑289 (MQ=255)`  
                                                                                                                                                         `gACAGCATTAAGTCCATTGGCGCGAAGGCTGGTATGACCGAAGAGGATATTATCAAGCAGGGCCAAATGGCTCGCATCCTACGGTTCAACGAGTACAACTTTATTGACAAGGAGATTTACCTGTGGAGACCGTAGCGTATATTGGTCCGGGTCTTTGTGTTCTCGGAGTGTGCCTCATTTCGTGGGGCCTTTGGGACTTAGCCAGAATAATCAAGTCGTTACACGACACTAAGTGATAAACTCAAGGTCCCTAAATTAATACGACTCACTATAGGGAGATAGGGGCCTTTACGATTATTAc                                                                                                                                                  <  1:230632/301‑1 (MQ=255)`  
                                                                                                                                                             `gCATTAAGTCCATTGGCGCGAAGGCTGGTATGACCGAAGAGGATATTATCAAGCAGGGCCAAATGGCTCGAATCCTACGGTTCAACGAGTACAACTTTATTGACACGGAGATTTACCTGTGGAGACCGTAGCGTATCTTGGTCCGGGTCTTTGTGTTCTCGGAGTGTGCCTCATTTCGTGGGGCCTTTGGGACTTAGCCAGAATAATCAAGTCGTTACACGACACTAAGTGATAAACTCAAGGTCCCTCAATTCATACGACTCACTATAGGGAGATAGGGGCATTTCCGCTTATTACtttt                                                                                                                                              >  1:293854/1‑300 (MQ=255)`  
                                                                                                                                                                    `gTCCATTGGCGCGAAGGCTGGTATGACCGAAGAGGATATTATCAAGCAGGGCCAAATGGCTCGAATCCTACGGTTCAACGAGTACAACTTTATTGACAAGGAGATTTACCTGTGGAGACCGTAGCGTATATTGGTCCTGGTCTTTGTGTTCTCGGAGTGTGCCTCATTTCGTGGGGCCTTTGGGACTTAGCCAGAATAATCAAGTCGTTACACGACACTAAGTGATAAACTCAAGGTCCCTAAATTAATACGACTCACTATAGGGAGATCGGGGCCTTTACGATTATTACTTTAAGATTTa                                                                                                                                       >  1:791137/1‑301 (MQ=255)`  
                                                                                                                                                                    `gTCCATTGGCGCGAAGGCTGGTATGACCGAAGAGGATATTATCAAGCAGGACCAAATGGCTCGAATCCTACGGTTCAACGAGTCCAACTTTATTGACAAGGAGATTTACCTGTGGAGACCGTAGCGTATATTTGTCCGGGTCTTTGTGTTCTCGGAGTGTGCCTCCTTTCGTGGGGCCTTTGGGACTTAGCCAGAATAATCAAGTCGTTACACGACACTAAGTGCTAAACTCAAGGTCCCTAAATTAATACGACTCACTATAGGGAGATAGTGGCCTTTACGATTCTTACTTTAAGCtttt                                                                                                                                       >  1:819014/1‑300 (MQ=255)`  
                                                                                                                                                                              `tgtataagagacaggACCGAAGAGGATATTATCAAGCAGGGCCAAATGGCTCGAATCCTACGGTTCAACGAGTACAACTTTATTGACAAGGAGATTTACCTGTGGAGACCGTAGCGTATATTGGTCCGGGTCTTTGTGTTCTCGGAGTGTGCCTCATTTCGTGGGGCCTTTGGGACTTAGCCAGAATAATCAAGTCGTTACACGACACTAAGTGATAAACTCAAGGTCCCTAAATTAATACGACTCACTATAGGGAGATAGGGGCCTTTACGATTATTACTTTAAGATTTAACTCTAagag                                                                                                                             <  1:507861/287‑1 (MQ=255)`  
                                                                                                                                                                                       `ggTATGACCGAAGAGGATATTATCAAGCAGGGCCAAATGGCTCGAATCCTACGGTTCAACGAGTACAACTTTATTGACAAGGAGATTTACATGTGGAGACCGTAGCGTATATTGGTCCGGGTCTTTGTGTTCTCGGAGTGTGCCTCATTTCGTGGGGCCTTTGGGACTTAGCCAGAATAATCAAGTCGTTACACGACACTAAGTGATAAACTCAAGGTCCCTAAATTAATACGACTCACTATAGGGAGATAGGGGCCTTTACGATTATTACTTTAAGATTTAACTCTAAGAGGAATCtttt                                                                                                                    >  1:202113/1‑300 (MQ=255)`  
                                                                                                                                                                                            `gACCGAAGAGGATATTATCAAGCAGGGCCAAATGGCTCGAATCCGACGGTTCAACGAGTACAACTTTATTGACAAGGAGATTTACCTGTGGAGACCGTAGCGTATATTGGTCCGGGTCTTTGTGTTCTCGGAGTGTGCCTCATTTCGTGGGGCCTTTGGGACTTAGCCAGAATAATCAAGTCGTTACACGACACTAAGTGATAAACTCAAGGTCCCTAAATTAATACGACTCACTATAGGGAGATAGGGGCCTTTACGATTATTACTTTAAGATTTAACTCTAAGAGGAATCTTTATTATg                                                                                                               <  1:618054/301‑1 (MQ=255)`  
                                                                                                                                                                                             `aCCGAACAGGATATTATCAAGCAGGGCCAAATTGCTCGAATCCTACGGTTCAACGAGTACAACTTTATTGACAAGGAGATTTACCTGTGGAGACCGTAGCGTATATTGGTCCGGGTCTTTGTGTTCTCGGAGTGTGCCTCATTTCGTGGGGCCTTTGGGACTTAGCCAGAATAATCAAGTCGTTACACGACACTAAGTGATAAACTCAAGGTCCCTAAATTAATACGACTCACTATAGGGAGATAGGGGCCTTTACGAATATTACTTTAAGATTTAACTCTAAGAGGAATCTTTATTATGt                                                                                                              >  1:457043/1‑301 (MQ=255)`  
                                                                                                                                                                                                   `gagGATATTATCAAGCAGGGCCAAATGGCTCGAATCCTACGGTTCAACGTGTACAACTTTATTGACAAGGAGATTTACCTGTGGAGACCGTAGCGTATATTGGTCCGGGTCTTTGTGTTCTCGGAGTGTGCCTCATTTCGTGGGGCCTTTGGGACTTAGCCAGAATAATCAAGTCGTTACACGACACTAAGTGATAAACTCAAGGTCCCTAAATTAATACGACTCACTATAGGGAGATAGGGGCCTTTACGATTATTACTTTAAGATTTAACTCTAAGAGGAATCTTTATTATGTTAacac                                                                                                        <  1:479006/301‑1 (MQ=255)`  
                                                                                                                                                                                                     `ggATATTATCAAGCAGGGCCAAATGGCTCGAATCCTACGGTTTACCGAGTACAACTTTATTGACAAGGAGATTTACCTGTGGAGACCGTAGCGTATATTGGTCCGGGTCTTTGTGTTCTCGGAGTGTGCCTCATTTCGTGGGGCCTTTGGGACTTAGCCAGAATAATCAAGTCGTGACACGACACTAAGTGATAAACTCAAGGTCCCTAAACTAATACGACTCACTATAGGGAGATAGGGGCCTTTACGATTATTACTTTAAGATTTAACTCTAAGAGGAATCTTtattatttgaagccct                                                                                                      >  1:365063/1‑291 (MQ=255)`  
                                                                                                                                                                                                     `ggATATTATCAAGCAGGGCCAAATGGCTCGAATCCTACGGTTCAACGAGTACAACTTTATTGACAAGGAGATTTACCTGTGGAGACCGTAGCGTATATTGGTCCGGGTCTTTGTGTTCTCGGAGTGTGCCTCATTTCGTGGGGCCTTTGGGACTTAGCCCGAATAATCAAGTCGTTACACGACACTAAGTGATAAACTCAAGGTCCCTAAATTAATACCACTCACTATCGGGAGATAGGGGCCTTTACGATTATTACTTTAAGATTTAACTATAAGAGGAATCTTTATTATGTTAACACCt                                                                                                      >  1:152907/1‑301 (MQ=255)`  
                                                                                                                                                                                                     `ggATATTATCAAGCAGGGCCAAATGGCTCGAATCCTACGGTTCAACGAGTACAACTTTATTGACAAGGAGATTTACCTGTGGAGACCGTAGCGTATATTGGTCCGGGTCTTTGTGTTCTCGGAGTGTGCCTCATTTCGTGGGGCCTTTGGGACTTAGCCAGAATAATCAAGTCGTTACACGACACTAAGTGATAAACTCAAGGTCCCTAAATTAATACGACTCACTATAGGGAGATAGGGGCCTTTACGATTATTACTTTAAGATTTAACTCTAAGAGGAATCTTTTTTATGTTactgtct                                                                                                      >  1:247482/1‑295 (MQ=255)`  
                                                                                                                                                                                                           `aaagagacAGGGCCAAATGGCTCGAATCCTACGGGTCAACGAGTACAACTTTATTGACAAGGAGATTTACCTGTGGAGACCGTAGCGTATATTGGGCCGGGTCTTTGTGTTCTCGGAGTGTGCCTCATTTCGTGGGGCCTTTGGGACTTAGCCAGAATAATCAAGTCGTTACACGACACTAAGTGATAAACTCAAGGTCCCTAAATTAATACGACTCACTATAGGGAGATAGGGGCCTTTACGATTATTACTTTAAGATTTAACTCTAAGAGGAATCTTTATTATGTTAACACCTATTAAc                                                                                                <  1:731134/294‑1 (MQ=255)`  
                                                                                                                                                                                                                          `aaTGGCTCGAATCCTACGGTTCAACGAGTACAACTTTATTGACAAGGAGATTTACCTGTGGAGACCGTAGCGTATATTGGTCCGGGTCTTTGTGTTCTCGGAGTGTGCCTCTTTTCGTGGGGCCTTTGGGACTTAGCCAGAATAATCAAGTCGTTACACGACACTAAGTGATAAACTCAAGGTCCCTAAATTAATACGACTCACTATAGGGAGATAGGGGCCTTTACGATTATTACTTTAAGATTTAACTCTAAGAGGAATCTTTATTATGTTAACACCTATTAACCAATTACTTAAGAAc                                                                                 <  1:449511/301‑1 (MQ=255)`  
                                                                                                                                                                                                                                 `cGAATCCTACGGTTCAACGAGTACAACTTTATTGACAAGGATATTTACCTGTGGAGACCGTAGCGTATATTGGTCCGGGTCTTTGTGTTCTCGGAGTGTGCCTCATTTCGTGGGGCCTTTGGGACTTAGCCAGAATAATCAAGTCGTTACACGACACTAAGTGATAAACTCAAGGTCCCTAAATTAATACGACTCACTATAGGGAGATAGGGGCCTTTACGATTATTACTTTAAGATTTAACTCTAAGAGGAATCTTTATTATGTTAACACCTATTAACCAATTACTTAAGAACCCTAACg                                                                          <  1:400021/301‑1 (MQ=255)`  
                                                                                                                                                                                                                                   `aaTCCTACGGTTCAACGAGTACAACTTTATTGACAAGGAGATTTACCTGTGGAGACCGTAGCGTATATTGGTCCGGGTCTTTGTGTTCTCGGAGTGTGCCTCATTTCGTGGGGCCTTTGGGACTTAGCCAGAATAATCAAGTCGTTACACGACACTAAGTGATAAACTCAAGGTCCCTAAATTAATACGACTCACTATAGGGAGATAGGAGCCTTTACGATTATTACTTTAAGATTTAACTCTAAGAGGAATCTTTATTATGTTAACACTTATTAACCAATTACTTAAGAACCCTAACGaa                                                                        >  1:94079/1‑300 (MQ=255)`  
                                                                                                                                                                                                                                      `ccTACGGTTCAACGAGTACAACTTTATTGACAAGGAGATTTACCTGTGGAGACCGTAGCGTATATTGGTACGGGGCTTTGTGTTCTCGGAGTGTGCCTCATTTCGTGGGGCCTTTGGGACTTAGCCAGAATAATCAAGTCGTTACACGACACTAAGTGATAAACTCAAGGTCCCTAAATTAATACGACTCACTATAGGGAGATAGGGGCCTTTACGATTATTACTTTAAGATTTAACTCTAAGAGGAATCTTTATTATGTTACCACCTATTAACCAATTACTTAAGAACACTAACGATAtt                                                                     >  1:500577/1‑301 (MQ=255)`  
                                                                                                                                                                                                                                           `ggTTCAACGAGTACAACTTTATTGACAAGGAGATTTACCTGTGGAGACCGTAGCGTATATTGGTCCGGGTCTTTGTGTTCTCGGAGTGTGCCTCATTTCGTGGGGCCTTTGGGACTTAGCCAGAATAATCAAGTCGTTACACGACACTAAGTGATAAACTCAAGGTCCCTAAATTAATACGACTCACTATAGGGAGATAGGGGCCTTTACGATTATTACTTTAAGATTTAACTCTAAGAGGAATCTTTATTATGTTAACACCTATTAACCAATTACTTAAGAACCCTAACGATATTCCAGa                                                                >  1:337142/1‑301 (MQ=255)`  
                                                                                                                                                                                                                                             `ttCAACGAGTACAACTTTATTGACAAGGAGATTTACCTGTGGAGACCGTAGCGTATATTGGTCCGGGTCTTTGTGTTCTCGGAGTGTGCCTCATTTCGTGGGGCCTTTGGGACTTAGCCAGAATAATCAAGTCGTTACACGACACTAAGTGATAAACTCAAGGTCCCTAAATTAATACGACTCACTATAGGGAGATAGGGGCCTTTACGATTATTACTTTAAGATTTAACTCTAAGAGGAATCTTTATTATGTTAACACCTATTAACCAATTACTTAAGAACCCTAACGATAATCCAGATg                                                              <  1:136575/301‑1 (MQ=255)`  
                                                                                                                                                                                                                                             `aaaaaagAGTACAACATTATGGACAAGGAGATTTAACTGTGGAGACCGTAGAATATATTGGTCAGGGTCTTGGTGATCTCGGAGTGTGCCTCATGTCGTGGGGCCTTTGGGAAATAGACAGAATAAACAAGACATTACAAGAAACTAAGTGATAAACTAAAGGTCCCAAAAGTAATACTACAAAATAAAGGGAGATAGGGGACTTTACGATAATTACTTTAAGAAATAAATCTAAGAGGAATCTTTATTATGTTAACACCTATTAACCAATTACTTAAGAACCCTAACGATATTCCAGATg                                                              <  1:655755/295‑1 (MQ=255)`  
                                                                                                                                                                                                                                               `cAACGAGTACAACTTTATTGACAAGGAGATTTACCTGTGGAGACCGTAGCGTATATTGGTCCGGGTCTTTGTGTTCTCGGAGTGTGCCTCATTTCGTGGGGCCTTTGGGACTTAGCCAGAATAATCAAGTCGTTACACGACACTAAGTGATAAACTCAAGGTCCCTAAATTAATACGACTCACTATAGGGAGATAGGGGCCTTTACGATTATTACTTTAAGATTTAACTCTAAGAGGAATCTTTATTATGTTAACACCTATTAA‑CAATTACTTAAGAACCCTAACGATATTCCAGATGTAc                                                           <  1:357497/301‑1 (MQ=255)`  
                                                                                                                                                                                                                                                   `gAGTACAACTTTATTGACAAGGAGATTTACCTGTGGAGACCGTAGCGTATATTGGTCCGGGTCTTTGTGTTCTCGGAGTGTGCCTCATTTCGTGGGGCCTTTGGGACTTAGCCAGAATAATCAAGTCGTTACACGACACTAAGTGATAAACTCAAGGTCCCTAAATTAATACGACTCACTATAGGGAGATAGGGGCCTTTACGATTATTACTTTAAGATTTAACTCTAAGAGGAATCTTTATTATGTTAACACCTATTAACCAATTACTTAAGAACCCTAACGAAAATCCAGAAGGACCTc                                                        >  1:521709/1‑301 (MQ=255)`  
                                                                                                                                                                                                                                                     `gTACAACTTTATTGACAAGGAGATTTACCTGTGGAGACCGTAGCGTATATTGGTCCGGGTCTTTGTGTTCTCGGAGTGTGCCTCATTTCGTGGGGCCTTTGGGACTTAGCCAGAATAATCAAGTCGTTACACGACACTAAGTGATAAACTCAAGGTCCCTAAATTAATACGACTCACTATAGGGAGATAGGGGCCTTTACGATTATTACTTTAAGATTTAACTCTAAGAGGAATCTTTATTATGTTAACACCTATTAACCAATTACTTAAGAACCCTAACGactgtctcttatacacatct                                                      >  1:217029/1‑282 (MQ=255)`  
                                                                                                                                                                                                                                                     `gTACAACTTTATTGACAAGGAGATTTACCTGTGGAGACCGTAGCGTATATTGGTCCGGGTCTTTGTGTTCTCGGAGTGTGCCTCATTTCGTGGGGCCTTTGGGACTTAGCCAGAATAATCAAGTCGTTACACGACACTAAGTGATAAACTCAAGGTCCCTAAATTAATACGACTCACTATAGGGAGATAGGGGCCTTTACGATTATTACTTTAAGATTTAACTCTAAGAGGAATCTTTATTATGTTAACACCTATTAACCAATTACTTAAGAACCCTAACGATATTCCAGATGTACCTCGt                                                      >  1:46363/1‑301 (MQ=255)`  
                                                                                                                                                                                                                                                                 `tGACAAGGAGATTTACCTGTGGAGACCGTAGCGTATATTGGTCCGGGTCTTTGTGTTATCGGAGTGTGCCTCATTTCGTGGGGCCTTTGGGACTTAGCCAGAATAATCAAGTCGTTACACGACACTAAGTGATAAACTCAAGGTCCCTAAATTAATACGACTCACTATAGGGAGATAGGGGCCTTTACGATTATTACTTTAAGATTTAACTCTAAGAGGAATCTTTATTATGTTAACACCTATTAACCAATTACTTAAGAACCCTAACGATATTCCAGATGTACCTCGTGCAACCGCTGAg                                          <  1:747803/301‑1 (MQ=255)`  
                                                                                                                                                                                                                                                                 `gatgtgtataagagacaggtgGAGACCGTAGCGTATATTGGTCCGGGTCTTTGTGTTCTCGGAGTGTGCCTCATTTCGTGGGGCCTTTGGGACTTACCCAGAATAATCAAGTCGTTACACGACACTAAGTGATAAACTCAAGGTCCCTAAATTAATACGACTCACTATAGGGAGATAGGGGCCTTTACGATTATTACTTTAAGATTTAACTCTAAGAGGAATCTTTATTATGTTAACACCTATTAACCAATTACTTAAGAACCCTAACGATATTCCAGATGTACCTCGTGCAACCGCTGAg                                          <  1:385788/283‑1 (MQ=255)`  
                                                                                                                                                                                                                                                                        `gtgtataagagacagagaCCGTAGCGTATATTGGTCCGGGTCTTTGTGTTCTCGGAGTGTGCCTCATTTCGTGGGGCCTTTGGGACTTAGCCAGAATAATCAAGTCGTTACACGACACTAAGTGATAAACTCAAGGTCCCTAAATTAATACGACTAACTATAGGGAGATAGGGGCCTTTACGATTATTACTTTAAGATTTAACTCTAAGAGGAATCTTTATTATGTTAACACCTATTAACCAATTACTTAAGAACCCTAACGATATTCCAGATGTACCTCGTGCAACCGCTGAGTATCTAc                                   <  1:287215/287‑1 (MQ=255)`  
                                                                                                                                                                                                                                                                        `gagaTTTACCTGTGGAGGCCGTCGCGTATATTGGTCCGGGTCTTTGTGTTCTCGGAGTGTGCCTCATTTCGTTGGGCCTTTGGGACTTAGCCAGAATAATCAAGTCGTTACACGACACTAAGTGATAAACTCAAGGTCCCTAAATTAATACGACTCACTATAGGGAGATAGGGGCCTTTACGATTATTACTTTAAGATTTAACTCTAAGAGGAATCTTTATTATGTTAACACCTATTAACCAATTACTTAAGAACCCTAACGATATTCCAGATGTACCTCGTGAAACCGCTGAGTATCTAc                                   <  1:350447/301‑1 (MQ=255)`  
                                                                                                                                                                                                                                                                        `aggaTTTACCTGTGGAGACCGTAGCGTATATTGGTCCGGGTCTTTGTGTTCTCGGAGTGTGCCTCATTTCGTGGGGCCTTTGGGACTTAGCCAGAATAATCAAGTCGTTACACGACACTAAGTGATAAACTCAAGGTCCCTAAATTAATACGACTCACTATAGGGAGATAGGGGCCTTTACGATTATTACTTTAAGATTTAACTCTAAGAGGAATCTTTATTATGTTAACACCTATTAACCAATTACTTAAGAACCCTAACGATATTCCAGATGTACCTCGTGCAACCGCTGAGTATCTAc                                   <  1:485494/299‑1 (MQ=255)`  
                                                                                                                                                                                                                                                                         `ataTTTACCTGTGGAGACCGTAGCGTATATTGGTCCGGGTCTTTGTGTTCTCGGAGTGTGCCTCATTTCGTGGGGCCTTTGGGACTTAGCCAGAATAATCAAGTCGTTACACGACACTAAGTGATAAACTCAAGGTCCCTAAATTAATACGACTCACTATAGGGAGATAGGTGCCTTTACGATTATTACTTTAAGATTTAACTATAAGAGGAATCTTTATTATGTTAACACCTATTAACCAATTACTTAAGAACCCTAACGATATTCCAGATGTACCTCGTGCAACCGCTGAGTATCTACa                                  <  1:553999/299‑1 (MQ=255)`  
                                                                                                                                                                                                                                                                         `agaTTTACCTGTGGAGACCGTAGCGTATATTGGTCCGGGTCTTTGTGTTCTCGGAGTGTGCCTCATTTCGTGGGGCCTTTGGGACTTAGCCAGAATAATCAAGTCGTTACACGACACTAAGTGATAAACTCAAGGTCCCTAAATTAATACGACTCACTATAGGGAGATAGGGGCCTTTACGATTATTACTTTAAGATTTAACTCTAAGAGGAATCTTTATTATGTTAACACCTACTAACCAATTACTTAAGAACCCTAACGATATTCCAGATGTACCTCGTGCAACCGCTGAGTATCTACa                                  <  1:7698/301‑1 (MQ=255)`  
                                                                                                                                                                                                                                                                          `gaTTTACCTGTGGAGACCGTAGCGTATATTGGTCCGGGTCTTTGTGTTCTCGGAGTGTGCCTCATTTCGTGGGGCCTTTGGGACTTAGCCAGAATAATCAAGTCGTTACACGACACTAAGTGATAAACTCAAGGTCCCTAAATTAATACGACTCACTATAGGGAGATAGGGGCCTTTACGATTATTACTTTAAGATTTAACTCTAAGAGGAATCTTTATTATGTTAACACCTATTAACCAATTACTTAAGAACCCTAACGATATTCCAGATGTACCTCGTGCAACCGCTGAGtctgtctct                                 >  1:378631/1‑293 (MQ=255)`  
                                                                                                                                                                                                                                                                          `gaTTTACCTGTGGAGACCGTAGCGTATATTGGTCCGGGTCTTTGTGTTCTCGGAGTGTGCCTCATTTCGTGGGGCCTTTGGGACTTAGCCAGAATAATCAAGTCGTTACACGACACTAAGTGATAAACTCAAGGTCCCTAAATTAATACGACTCACTATAGGGAGATAAGGGCCTTTACGATTATTACTTTAAGATTTAACTCTAAGAGGAATCTTTATTATGTTAACACCTATTAACCAATTACTTAAGAACCCTAACGATATTCCAGATGTACCTCGTGCAACCGcctgtctcttatac                                 >  1:705480/1‑288 (MQ=255)`  
                                                                                                                                                                                                                                                                             `ttACCTGTGGAGACCGTAGCGTATATTGGTCCGGGTCTTTGTGTTCTCGGAGTGTGCCTCATTTCGTGGGGCCTTTGGGACTTAGCCAGAATAATCAAGTCGTTACACGACACTAAGTGATAAACTCAAGGTCCCTAAATTAATACGACTCACTATAGGGAGATAGGGGCCTTTACGATTATTACTTTAAGATTTAACTATAAGAGGAATCTTTATTATGTTAACACCTATTAACCAATTACTTAAGAACCCTAACGATATTCCAGATGTACCTCGTGCAACCGCTGAGTATCTACAGGtt                              <  1:471717/301‑1 (MQ=255)`  
                                                                                                                                                                                                                                                                                   `gtgGAGACCGTAGCGTATATTGGTCCGGGTCTTTGTGTTCTCGGAGTGTGCCTCATTTCGTGGGGCCTTTGGGACTTAGCCAGAATAATCAAGTCGTTACACGACACTAAGTGATAAACTCAAGGTCCCTAAATTAATACGACTCACTATAGGGAGATAGGGGCCTTTACGATTATTACTTTAAGATTTAACTCTAAGAGGAATCTTTATTATGTTAACACCTATTAACCAATTACTTAAGAACCCTAACGATATTCCAGATGTACCTCGTGCAACCGCTGAGTATCTACAGGTTCGATTc                        >  1:665150/1‑301 (MQ=255)`  
                                                                                                                                                                                                                                                                                     `ggAGACCGTAGCGTATATTGGTCCGGGTCTTTGTGTTCTCGGAGTGTGCCTCATTTCGTGGGGCCTTTGGGACTTAGCCAGAATAATCAAGTCGTTACACGCCACTAAGTGATAAACTCAAGGTCCCTAAATTAATACGACTCACTATAGGGAGATAGGAGCCTTTACGATTATTACTTTAAGATTTAACTCTAAGAGGAATCTTTATTATGTTAACACCTATTAACCAATTACTTAAGAACCCTAACGATATTCCAGATGTACCTCGTGCAACCGCTGAGTATCTActgtctcttataca                      >  1:264859/1‑288 (MQ=255)`  
                                                                                                                                                                                                                                                                                      `gagaCCGTAGCGTATATTGGTCCGGGTCTTTGTGTTCTCGGAGTGTGCCTCATTTCGTGGGGCCTTTGGGACTTAGCCAGAATAATCAAGTCGTTACACGACACTAAGTGATAAACTCAAGGTCCCTAAATTAATACGACTCACTATAGGGAGATAGGGGCCTTTACGATTATTACTTTAAGATTTAACTCTAAGAGGAATCTTTATTATGTTAACACCTATTAACCAATTACTTAAGAACCCTAACGATATTCCAGATGTACCTCGTGCAACCGCTGAGTATCTACAGGTTCGATTCAAc                     <  1:424802/301‑1 (MQ=255)`  
                                                                                                                                                                                                                                                                                          `ccGTAGCGTATATTGGTCCGGGTCTTTGTGTTCTCGGAGTGTGCCTCATTTCGTGGGGCCTTTGGGACTTAGCCAGAATAATCAAGTCGTTACACGACACTAAGTGATAAACTCAAGGTCCCTAAATTAATACGACTCACTATAGGGAGATAGGGGCCTTTACGATTATTACTTTAAGATTTAACTCTAAGAGGAATCTTTATTATGTTAACACCTATTAACCAATTACTTAAGAACCCTAACGATATTCCAGATGTACCTCGTGCAACCGCTGAGTATCTACAGGTTCGATTCAACTAtt                 >  1:432597/1‑300 (MQ=255)`  
                                                                                                                                                                                                                                                                                           `cGTAGCGTATATTGGTCCGGGTCTTTGTGTTCTCGGAGTGTGCCTCATTTCGTGGGGCCTTTGGGACTTAGCCAGAATAATCAAGTCGTTACACGACACTAAGTGATAAACTCAAGGTCCCTAAATTAATACGACTCACTATAGGGAGATAGGGGCCTTTACGATTATTACTTTACGATTTAACTCTAAGAGGAATCTTTATTATGTTAACACCTATTAACCAATTACTTAAGAACCCTAACGATATTCCAGATGTACCTCGTGTAACCGCTCAGTATCTCCAGGTTCGATTCACCTAttc                >  1:224600/1‑299 (MQ=255)`  
                                                                                                                                                                                                                                                                                             `acaggTATATTGGTCCGGGTCTTTGTGTTCTCGGAGTGTGCCTCATTTCGTGGGGCCTTTGGGACTTAGCCAGAATAATCAAGTCGTTACACGACACTAAGTGATAAACTCAAGGTCCCTAAATTAATACGACTCACTATAGGGAGATAGGGGCCTTTATGATTATTACTTTAAGATTTAACTCTAAGAGGAATCTTTATTATGTTAACACCTATTAACCAATTACTTAAGAACCCTAACGATATTCCAGATGTACCTCGTGCAACCGCTGAGTATCTACAGGTTCGATTCAACTATGCGt              <  1:79808/297‑1 (MQ=255)`  
                                                                                                                                                                                                                                                                                              `aGCGTATATTGGTCCGGGTCTTTGTGTTCTCGGAGTGTGCCTCATTTCGTGGGGCCTTTGGGACTTAGCCAGAATAATCAAGTCGTTACACGACACTAAGTGATAAACTCAAGGTCCCTAAATTAATACGACTCACTATAGGGAGATAGGGGCCTTTACGATTATTACTTTAAGATTTAACTCTAAGAGGAATCTTTATTATGTTAACACCTATTAACCAATTACTTAAGAACCCTAACGATATTCCAGATGTACCTCGTGCAACCGCTGAGTATCTACAGGTTCGATTCAACTATGCGTa             >  1:740182/1‑301 (MQ=255)`  
                                                                                                                                                                                                                                                                                              `aGCGTATATTGGTCCGGGTCTTTGTGTTCTCGGAGTGTGCCTCATTTCGTGGGGCCTTTGGGACTTAGCCAGAATAATCAAGTCGTTACACGACACTAAGTGATAAACTCAAGGTCCCTAAATTAATACGACTCACTATAGGGAGATAGGGGCCTTTACGATTATTACTTTAAGATTTAACTCTAAGAGGAATCTTTATTATGTTAACACCTATTAACCAATTACTTAAGAACCCTAACGATATTCCAGATGTACCTCGTGCAACCGCTGAGTATCTACAGGTTCGATTCAACTATGCGTa             >  1:811854/1‑301 (MQ=255)`  
                                                                                                                                                                                                                                                                                              `aGCGTATATTGGTCCGGGTCTTTGTGTTCTCGGAGTGTGCCTCATTTCGTGGGGCCTTTGGGACTTAGCCAGAATAATCAAGTCGTTACACGACACTAAGTGATAAACTCAAGGTCCCTAAATTAATACGACTCACTATAGGGAGATAGGGGCCTTTACGATTATTACTTTAAGATTTAACTCTAAGAGGAATCTTTATTATGTTAACACCTATTAACCAATTACTTAAGAACCCTAACGATATTCCAGATGTACCTCGTGCAACCGCTGAGTATCTACAGGTTCGATTCAACTATGCGTa             >  1:210265/1‑301 (MQ=255)`  
                                                                                                                                                                                                                                                                                                 `gTATATTGGTCCGGGTCTTTGTGTTCTCGGAGTGTGCCTCATTTCGTGGGGCCTTTGGGACTTAGCCAGAATAATCAAGTCGTTACACGACACTAAGTGATAAACTCAAGGTCCCTAAATTAATACGACTCACTATAGGGAGATAGGGGCCTTTACGATTATTACTTTAAGATTTAACTCTAAGAGGAATCTTTATTATGTTAACACCTATTAACCAATTACTTAAGAACCCTAACGATATTCCAGATGTACCTCGTTCAACCGCTGAGTATCTACAGGTTCGATTCAACTATGCTTACCt          >  1:575211/1‑301 (MQ=255)`  
                                                                                                                                                                                                                                                                                                 `gTATATTGGTCCGGGTCTTTGTGTTCTCGGAGTGTGCCTCATTTCGTGGGGCCTTTGGGACTTAGCCAGAATAATCAAGTCGTTACACGACACTAAGTGATAAACTCAAGGTCCCTAAATTAATACGACTCACTATAGGGAGATAGGGGCCTTTACGATTATTACTTTAAGATTTAACTCTAAGAGGAATCTTTATTATGTTAACACCTATTAACCAATTACTTAAGAA‑CCTAACGATATTCCAGATGTACCTCATGCAACCGCTGAGTATCTACAGGTTCGATTAAACTactgtctctta         >  1:784879/1‑291 (MQ=255)`  
                                                                                                                                                                                                                                                                                                    `tatTGGTCCGGGTCTTTGTGTTCTCGGAGTGTGCCTCATTTCGTGGGGCCTTTGGGACTTAGCCAGAATAATCAAGTCGTTACACGACACTAAGTGATAAACTCAAGGTCCCTAAATTAATACGACTCACTATAGGGAGATAGGGGCCTTTACGATTATTACTTTAAGATTTAACTCTAAGAGGAATCTTTATTATGTTAACACCTATTAACCAATTACTTAAGAACCCTAACGATATTCCAGATGTACCTCGTGCAACCGCTGAGTATCTACAGGTTCGATTCAACTATGCGTACCTCGa       <  1:239600/301‑1 (MQ=255)`  

                                                                                                                                                                                                                                                                                                            `|`                                                                                                                                                                                                                                                                                                           
`GGGATTGCTGGATGGGGTGATACCGCCGAGGACTTCTTGAATAACCCGTTCATAACCGAGCCTAAAACGTCTGTGCTTAAGTCCGGTAAGAACAAAGGCCAAGAGGTTACTAAATGGGTTAAACGCGACCCTGAGCCTCATGAGACGCTTTGGGACTGCATTAAGTCCATTGGCGCGAAGGCTGGTATGACCGAAGAGGATATTATCAAGCAGGGCCAAATGGCTCGAATCCTACGGTTCAACGAGTACAACTTTATTGACAAGGAGATTTACCTGTGGAGACCGTAGCGTATATTGGTCTGGGTCTTTGTGTTCTCGGAGTGTGCCTCATTTCGTGGGGCCTTTGGGACTTAGCCAGAATAATCAAGTCGTTACACGACACTAAGTGATAAACTCAAGGTCCCTAAATTAATACGACTCACTATAGGGAGATAGGGGCCTTTACGATTATTACTTTAAGATTTAACTCTAAGAGGAATCTTTATTATGTTAACACCTATTAACCAATTACTTAAGAACCCTAACGATATTCCAGATGTACCTCGTGCAACCGCTGAGTATCTACAGGTTCGATTCAACTATGCGTACCTCGAAGCGT  >  NC_001604.1/18119‑18716`

  

Alignment Legend

Aligned base mismatch/match (shaded by quality score): `ATCG/ATCG` `< 3 ≤` `ATCG/ATCG` `< 11 ≤` `ATCG/ATCG` `< 23 ≤` `ATCG/ATCG` `< 35 ≤` `ATCG/ATCG` `< 38 ≤` `ATCG/ATCG`

Unaligned base: `atcg`    Masked matching base: `atcg`    Alignment gap: `‑`    Deleted base: `‑`
